# Effect of vitamin D supplementation on COVID-19 outcomes: an umbrella review of systematic reviews

**DOI:** 10.3389/fnut.2025.1559471

**Published:** 2025-06-13

**Authors:** Pavlo Petakh, Iryna Kamyshna, Iryna Halabitska, Oleksandr Kamyshnyi

**Affiliations:** ^1^Department of Biochemistry and Pharmacology, Uzhhorod National University, Uzhhorod, Ukraine; ^2^Department of Medical Rehabilitation, I. Horbachevsky Ternopil National Medical University, Ternopil, Ukraine; ^3^Department of Therapy and Family Medicine, I. Horbachevsky Ternopil National Medical University, Ternopil, Ukraine; ^4^Department of Microbiology, Virology and Immunology, I. Horbachevsky Ternopil National Medical University, Ternopil, Ukraine

**Keywords:** COVID-19, vitamin D supplementation, mechanical ventilation, hospital stay, clinical outcomes, immune modulation, randomized controlled trials

## Abstract

**Background:**

Vitamin D is suggested as a supportive therapy to reduce the severity of COVID-19 due to its immunomodulatory and anti-inflammatory effects. However, its effect on critical outcomes, such as ICU admissions and mortality, shows significant variation across randomized clinical trials and meta-analyses.

**Objectives:**

To summarize the influence of vitamin D supplementation on ICU admissions and mortality among COVID-19 patients.

**Methods:**

Overall, 21 eligible studies were retrieved using a comprehensive search from Scopus, PubMed, and Web of Science. A citation matrix was developed, revealing a Corrected Covered Area (CCA) of 0.54, indicating moderate overlap. Fixed-effects models were applied to data with low heterogeneity (ICU admissions: Q = 10.87, *p* = 0.33), while random-effects models were used for mortality outcomes (Q = 27.23, *p* = 0.006). Pooled odds ratios (OR) with 95% confidence intervals (CI) quantified the overall effects.

**Results:**

Vitamin D supplementation was associated with a significant 38% reduction in ICU admissions (OR = 0.62; 95% CI: 0.54–0.71) and a 33% reduction in mortality risk (OR = 0.67; 95% CI: 0.56–0.79). The benefit was pronounced in vitamin D-deficient populations, although heterogeneity in mortality outcomes highlighted variability across studies.

**Conclusion:**

While these findings suggest that vitamin D supplementation may help reduce ICU admissions and mortality among COVID-19 patients—particularly in those with vitamin D deficiency—the results should be interpreted with caution. The observed variability and potential confounding factors underscore the need for further large-scale, randomized controlled trials with standardized dosing protocols before definitive clinical recommendations can be made.

## 1 Introduction

The COVID-19 pandemic began in December 2019 in Wuhan, China, with the emergence of a novel coronavirus known as SARS-CoV-2. As declared by the World Health Organization (WHO), by March 2020, the virus had spread worldwide, reaching a critical global scale (1). This situation undoubtedly escalated into a public health emergency of international concern due to the virus’s high contagiousness (2). Initially, scientists and healthcare professionals thought that COVID-19 mainly impacted the respiratory system, resulting in interstitial pneumonia and acute respiratory distress syndrome (3–5). However, subsequent research has shown that, in addition to respiratory complications, COVID-19 can lead to a broad spectrum of disorders affecting various organs, either directly or indirectly associated with the infection (6–8).

The symptoms of COVID-19 vary from asymptomatic and mild cases, which do not require special medical care, to moderate and severe cases that necessitate hospitalization, respiratory support, and even intensive care unit (ICU) treatment (9–11). Several risk factors are associated with the progression of the disease (12, 13). For example, elderly individuals are at the greatest risk of adverse outcomes and complications. Moreover, the likelihood of complications increases in patients with comorbidities such as cardiovascular diseases, diabetes, cancer, or obesity (14–18). Other studies have shown that factors like age, sex, race, obesity, diabetes, and hypertension play significant roles in triggering an uncontrolled release of cytokines, leading to disease exacerbation and an unbalanced immune response (19–24).

Since viral infections primarily spread through close social contact and large gatherings, nearly every country implemented social distancing measures to reduce the transmission of SARS-CoV-2 (25, 26). These measures aimed to limit the frequency of social interactions and increase physical distance between individuals, thereby decreasing the risk of human-to-human transmission (27). It has been demonstrated that staying at home for extended periods made people more prone to physical inactivity, unhealthy eating habits, and limited sunlight exposure, which could contribute to the development of vitamin D deficiency or insufficiency (28–30).

The active form of vitamin D [1,25(OH)2D3 — calcitriol] is a fat-soluble hormone that possesses numerous biological properties (endocrine, paracrine, and intracrine) in the human body. Its paracrine and intracrine functions have garnered significant interest, particularly due to the almost ubiquitous expression of the vitamin D receptor (VDR) by immune cells, highlighting its role in regulating acute and chronic inflammatory responses (31).

Several studies have demonstrated a correlation between vitamin D levels, age, oxygen therapy needs, and mortality (32–34). Daneshkhah et al. showed that COVID-19 mortality was highest in Italy, Spain, and France, European countries with the highest rates of severe vitamin D deficiency (35). Langlois et al. found that low vitamin D levels were associated with an increased frequency of infections, sepsis, and mortality (36). Other studies have established connections between inflammatory markers and disease progression. For instance, Ai-Ping Yang et al. demonstrated that white blood cell count, lymphocyte count, neutrophil count, C-reactive protein (CRP) levels, and ratios such as neutrophil-to-lymphocyte, lymphocyte-to-monocyte, and platelet-to-lymphocyte were statistically higher in patients with severe cases than in those with mild cases (37). Guohui et al. investigated novel serological markers in COVID-19 and, through multivariate logistic regression analysis, found that the CRP-to-albumin and CRP-to-prealbumin high-sensitivity ratios correlated with the risk of severe COVID-19 (38).

This systematic review and meta-analysis aimed to assess the effects of vitamin D supplementation on critical COVID-19 outcomes, including ICU admissions and mortality.

## 2 Materials and methods

### 2.1 Study design and data extraction

This meta-analysis was performed in order to determine the impact of vitamin D supplementation on severe cases of critical COVID-19 (i.e., ICU admission and mortality). Eligible studies included systematic reviews and meta-analyses investigating the association between vitamin D supplementation and COVID-19 severity.

A comprehensive literature search was performed on PubMed, Scopus, and Web of Science (WoS) on 20 December 2024. The following search strategies were used:

For PubMed, the search query used was: (Vitamin D OR Vit D) AND (COVID-19 OR SARS-CoV-2 OR COVID-2019 OR 2019-nCoV OR “2019 novel coronavirus infection” OR “coronavirus disease-19” OR “coronavirus disease 2019” OR “novel coronavirus”) AND (systematic review[pt] OR meta-analysis[pt] OR “systematic review”[tiab] OR “meta analysis”[tiab] OR “systematic overview”[tiab]) AND (supplementation OR supplement[tiab]).

For Scopus, the search query was: TITLE-ABS-KEY(“Vitamin D” OR “Vit D”) AND TITLE-ABS-KEY(COVID-19 OR SARS-CoV-2 OR COVID-2019 OR 2019-nCoV OR “2019 novel coronavirus infection” OR “coronavirus disease-19” OR “coronavirus disease 2019” OR “novel coronavirus”) AND TITLE-ABS-KEY (“systematic review” OR “meta analysis” OR “systematic overview”) AND TITLE-ABS-KEY(supplementation OR supplement).

For Web of Science (WoS), the search query was: TS = (“Vitamin D” OR “Vit D”) AND TS = (COVID-19 OR SARS-CoV-2 OR COVID-2019 OR 2019-nCoV OR “2019 novel coronavirus infection” OR “coronavirus disease-19” OR “coronavirus disease 2019” OR “novel coronavirus”) AND TS = (“systematic review” OR “meta analysis” OR “systematic overview”) AND TS = (supplementation OR supplement). Data extraction was performed independently by two reviewers using a standardized data collection form. Extracted data included the number of studies within each systematic review, the total sample size, primary outcomes (ICU admissions and mortality), and effect size measures such as odds ratios (OR) with corresponding 95% confidence intervals (CI). Any disagreements were resolved through discussion or by consulting a third reviewer.

### 2.2 Inclusion and exclusion criteria

For this umbrella review, only systematic reviews and meta-analyses that quantitatively synthesized the effect of vitamin D supplementation on COVID-19 outcomes were included. Eligible reviews evaluated individuals diagnosed with COVID-19, regardless of disease severity, and incorporated data from randomized controlled trials (RCTs) as well as reviews that combined RCTs with observational studies. Studies had to report primary outcome data for ICU admissions and/or mortality; when available, baseline vitamin D status was also considered.

Reviews were excluded if they did not focus on vitamin D supplementation or if they lacked a quantitative synthesis (e.g., narrative reviews or single observational studies). Additionally, any reviews that did not report data on ICU admissions or mortality, or were published in languages other than English, were excluded. In cases where overlapping primary studies were present across multiple reviews, all eligible reviews were initially considered, with overlapping data noted and carefully managed during analysis.

The review by Rawat et al (82) was excluded from the sensitivity analysis because it had a limited sample size—incorporating only five studies—and exhibited methodological inconsistencies that produced effect sizes significantly different from those in other reviews. Including this review risked disproportionately skewing the overall pooled estimates. Sensitivity analyses confirmed that excluding (82). improved the consistency of the results without altering the overall direction of the effect.

### 2.3 Quality assessment of included reviews

To assess the quality of the included reviews, the AMSTAR 2 (A Measurement Tool to Assess Systematic Reviews) was used (39, 40). This tool assesses methodological rigor based on several criteria, including the comprehensiveness of the search strategy, clear inclusion criteria, robust data extraction methods, and an adequate assessment of risk of bias. Reviews were classified as “high,” “moderate,” or “low” quality based on these assessments.

### 2.4 Statistical analysis

Pooled odds ratios (OR) and 95% confidence intervals (CI) were computed for ICU admissions and mortality to determine the overall effect of vitamin D supplementation. For ICU admissions, heterogeneity was low (Q = 10.87, *p* = 0.33), so a fixed-effects model was applied using the inverse-variance method. In contrast, for mortality, significant heterogeneity (Q = 27.23, *p* = 0.006) warranted a random-effects model (DerSimonian and Laird) to account for between-study variability.

#### 2.4.1 Heterogeneity assessment

Heterogeneity among the studies was evaluated using Cochran’s Q-statistic and quantified by the I^2^ statistic, which represents the percentage of total variation across studies attributable to heterogeneity rather than chance. An I^2^ value of 25, 50, and 75% was interpreted as low, moderate, and high heterogeneity, respectively.

#### 2.4.2 Meta-regression analysis

A meta-regression analysis was performed using the logarithm of the odds ratio (log_OR) as the dependent variable and the publication year as the independent variable. The weighted least squares (WLS) method was employed, where inverse variance weighting was used to account for differences in study precision. Bubble plots were generated to visually examine the relationship between study year and effect size. The size of each bubble represents the weight assigned to each study, reflecting the study’s precision (inverse of variance).

#### 2.4.3 Calculation of pooled estimates

For each outcome of interest, we calculated pooled odds ratios (ORs) using a weighted average of individual study estimates, with weights assigned based on the inverse of each study’s variance. The tau-squared (τ^2^) statistic was used to quantify between-study variance, while statistical significance of the pooled ORs was assessed via Z-tests (*p* < 0.05 was considered significant).

To evaluate the stability of our results, we conducted a sensitivity analysis using a leave-one-out approach. In this procedure, each systematic review was removed one at a time, and the overall effect was recalculated. We compared these new pooled estimates, along with heterogeneity metrics (Q and I^2^), to the original values to determine whether any single review had a disproportionate impact on the findings.

One review, conducted by Rawat et al. (82) and Meng et al. (21), was flagged as an outlier due to its small sample size and methodological inconsistencies. Its effect estimates deviated substantially from those of the other reviews. When Rawat et al. (82) was excluded, the recalculated ORs for ICU admission and mortality remained consistent, suggesting that our main results are robust and not unduly affected by individual studies.

#### 2.4.4 Software and visualization

All statistical analyses were conducted using Python (v3.9) and relevant libraries (numpy, scipy, and matplotlib). Forest plots were generated to visually represent the pooled effect sizes and confidence intervals. The shaded area on the forest plot reflects the 95% confidence interval for the pooled OR, while vertical lines indicate the null effect (OR = 1).

## 3 Results

### 3.1 Study selection

The systematic search yielded a total of 402 records from Scopus (*n* = 213), PubMed (*n* = 82), and Web of Science (*n* = 107). After the removal of 213 duplicate records, 189 unique records proceeded to title and abstract screening. Following this stage, 65 records were excluded based on irrelevance to the research question or failure to meet the inclusion criteria. Full-text retrieval was attempted for the remaining 124 studies, with 10 records unavailable despite comprehensive efforts to obtain them.

**Figure 1:**
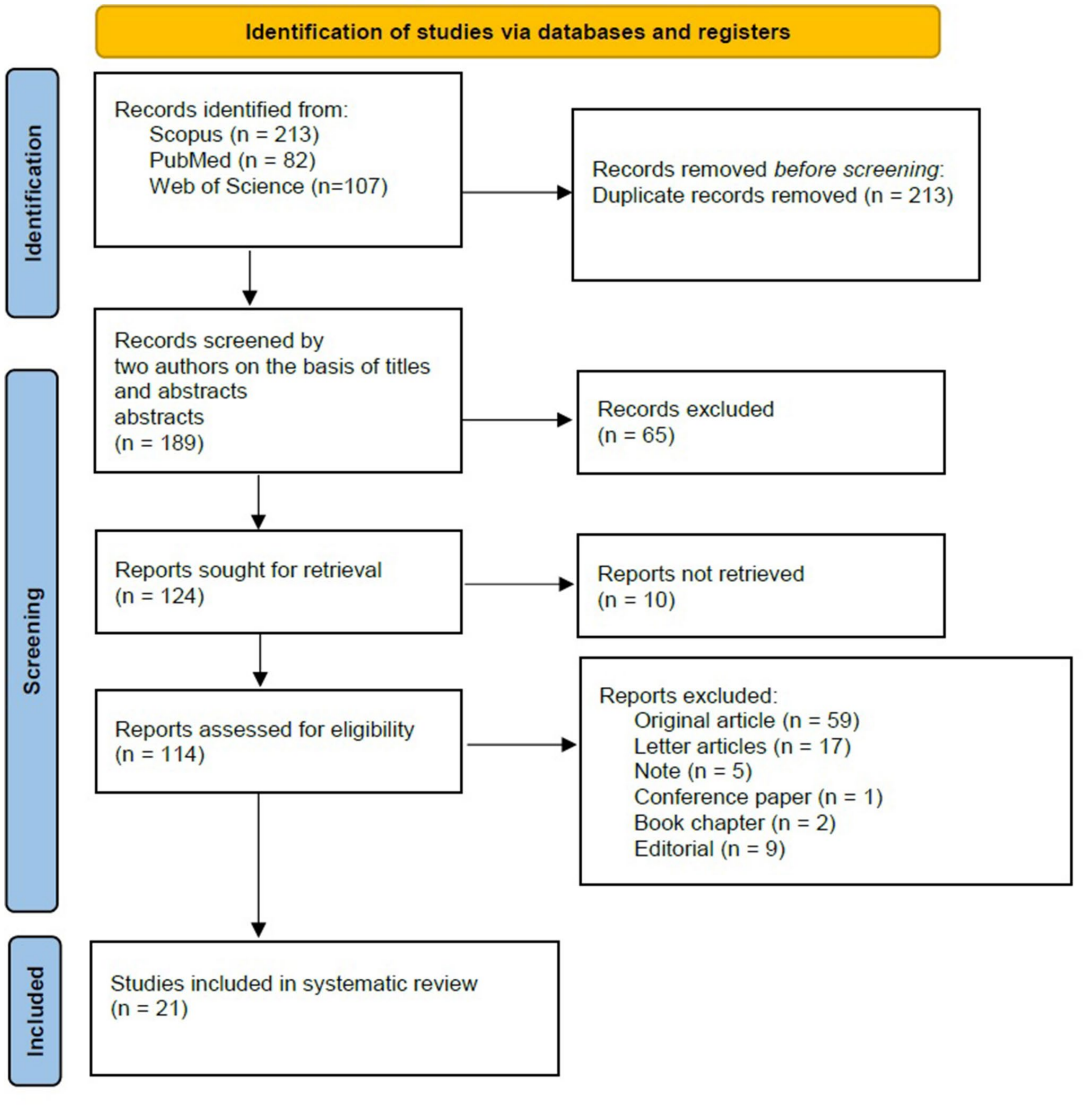
RISMA flow diagram for study selection. This flow diagram outlines the process of study identification, screening, eligibility assessment, and inclusion in the systematic review and meta-analysis. A total of 402 records were initially identified through database searches. After the removal of duplicates and irrelevant records, 21 studies met the inclusion criteria and were analyzed. Reasons for exclusion at each stage are detailed, including non-retrievable reports and studies that did not meet eligibility criteria.

A detailed eligibility assessment was conducted for 114 full-text articles, resulting in the exclusion of 93 studies for the following reasons: Ultimately, 21 studies met the predefined eligibility criteria and were included in the final systematic review and meta-analysis (Figure 1; Table 1).

**Table 1:** Characteristics of included studies.

References	Search strategy	No. of studies included	Total no. of participants	Dose regimen and period	Outcomes	Results
Sartini et al. (43)	PubMed/MEDLINE, Scopus, Cochrane, and Google Scholar	29	Not specified	Vitamin D dosages varied considerably, including daily, weekly, and monthly doses. Example regimens: 80,000 IU/day, 21,280 IU/day initial dose followed by 10,640 IU/day maintenance dose, or 200,000 IU/day.	Mortality, ICU admissions, intubation rates, and hospital length of stay (LOS).	Significant reduction in ICU admissions (OR = 0.55, 95% CI: 0.37–0.79), intubation rates (OR = 0.50, 95% CI: 0.27–0.92). Mortality reduction in analytical studies (OR = 0.45, 95% CI: 0.24–0.86). LOS showed non-significant reduction (−0.62 days). Subgroup effects in older and severe COVID-19 cases.
Zhang et al. (47)	PubMed, Embase, Web of Science, and Cochrane	21	4,553	Single-dose vs. continuous-dose; total intake within 14 days (≥100,000 IU vs. <100,000 IU). Baseline serum Vitamin D levels compared (deficient vs. non-restricted).	Mortality, ICU admissions, intubation rates, LOS.	Continuous dosing and lower doses (<100,000 IU) were more effective. Mortality and ICU admission rates were significantly reduced in the deficient group. No significant effect on LOS.
Adil et al. (42)	PubMed, Cochrane, CINAHL, EMBASE, and Google Scholar	14	2,165	Dosages ranged from 5,000 IU to 500,000 IU, administered daily for 1 day to 2 weeks.	ICU admissions, mechanical ventilation, mortality, hospital stay, oxygen requirement.	ICU admissions and need for mechanical ventilation reduced. No significant impact on mortality, hospital stay length, or oxygen requirement.
Ghoreshi et al. (53)	PubMed, Scopus, Web of Science, Embase, and Cochrane	16	The sample size in the evaluated studies ranged from 40 to 237 patients.	Dosage varied from 2000 to 500,000 IU.	Hospital LOS, CRP, ferritin, D-dimer, Hb, lymphocyte counts.	Reduced LOS (MD = −1.16; *p* = 0.033), especially with ≤10,000 IU doses. Significant CRP reduction in older adults. No significant changes in other inflammatory markers.
Yang et al. (50)	Cochrane Library, PubMed, Web of Science, and Embase	19	2,345 participants	Single-dose vs. multiple-dose administration.	ICU admission, mechanical ventilation (MV), LOS, mortality, inflammatory markers.	Multiple-dose regimens showed greater reductions in ICU admissions (OR 0.39), MV (OR 0.18), and LOS. No significant mortality or inflammatory marker changes.
Sobczak and Pawliczak (48)	PubMed, Web of Science, Embase, and Cochrane Central Register of Controlled Trials	13	Not specified	Not specified.	ICU admissions, mortality.	ICU admissions reduced (RR 0.73; *p* = 0.02). Mortality significantly lowered (RR 0.56; *p* = 0.02).
Jamilian et al. (72)	Web of Science, PubMed, Scopus, and Embase	13	Not specified	Not specified.	Mortality, infection risk, disease severity.	Reduced mortality in intervention studies (ES = 0.42) and observational studies (ES = 1.99). Vitamin D deficiency increased infection risk and severity.
Meng et al. (49)	PubMed, Cochrane Library, Embase, Web of Science, and Google Scholar	29	8,128	Ranging from 800 IU to 5,000 IU	ICU admissions, mechanical ventilation, mortality, SARS-CoV-2 infection prevention.	ICU admissions and MV rates reduced. No conclusive preventive effect against SARS-CoV-2 infection.
Sîrbu et al. (73)	PubMed, Embase	13	Not specified	Not specified.	Length of hospital stay (LOS), ICU admissions, mortality	High-dose vitamin D showed potential benefits in reducing LOS and ICU admissions. No significant effect on mortality.
Cao et al. (74)	PubMed, Embase, Web of Science, Cochrane Library, Google Scholar	Not specified.	Not specified.	Not specified.	Mortality	All-cause mortality decreased (RR = 0.54, 95% CI: 0.33–0.88).
Zhang et al. (75)	PubMed, Embase, Cochrane Library, CBM, CNKI, VIP, and WanFang	16	3,359	Not specified.	Mortality, ICU admissions, LOS.	No significant reduction in mortality in RCTs (RR = 0.94, 95% CI: 0.69–1.29). Positive impact on mortality in cohort studies (RR = 0.33, 95% CI: 0.23–0.47). No significant differences in ICU admission or MV rates.
Zaazouee et al. (76)	PubMed, Embase, Scopus, Web of Science, and Cochrane Library	9	1,586	Not specified.	ICU admissions, inflammatory markers	Reduced ICU admissions.
Kümmel et al. (51)	PubMed, Embase, Scopus, Web of Science, The Cochrane Library, medRxiv, Cochrane COVID-19 Study Register, and ClinicalTrial.gov	8	657	Not specified.	Mortality, ICU admissions.	No significant effects, but a trend for reduced mortality (OR = 0.74, 95% CI: 0.32–1.71) and reduced ICU admissions (OR = 0.41, 95% CI: 0.15–1.12).
D’Ecclesii et al. (77)	PubMed, Ovid Medline, EMBASE, and ISI Web of Science	38	Not specified	Not specified	ICU admissions, mechanical ventilation, LOS.	Vitamin D supplementation associated with lower risk of severe COVID-19 (SRR = 0.38, 95% CI: 0.20–0.72) and mortality (SRR = 0.35, 95% CI: 0.17–0.70). Older individuals and higher latitudes showed greater reduction in mortality.
Hosseini et al. (44)	PubMed, Cochrane, CINAHL, and EMBASE	5	1548 (RCT) and 586,841 (NRIS)	Not specified.	COVID-19 infection risk, hospital admission, ICU admission, mortality	Reduced ICU admission (RR = 0.35, 95% CI: 0.20–0.62) and mortality (RR = 0.46, 95% CI: 0.30–0.70). No significant effect on infection risk
Beran et al. (52)	PubMed/MEDLINE, Embase, and Cochrane	Not specified.	Not specified.	Not specified.	ICU admissions, LOS.	Vitamin D did not reduce mortality (RR 0.75, 95% CI 0.49–1.17) but reduced intubation rate (RR = 0.55, 95% CI 0.32–0.97) and LOS (MD = −1.26; 95% CI − 2.27 to −0.25).
Tentolouris et al. (78)	Med, Google Scholar, Embase, Web of Science, and medRxiv	9	2078	Not specified.	Mortality, ICU admissions, mechanical ventilation	No significant effect on mortality (OR = 0.60, 95% CI: 0.32–1.12). ICU admissions significantly reduced (OR = 0.33, 95% CI: 0.15–0.71).
Stroehlein et al.(79)	Cochrane Library and PubMed	3	645	Not specified.	Mortality, LOS.	No significant effect on mortality. Limited data on LOS.
Szarpak et al. (80)	PubMed, EMBASE, Web of Science, Cochrane, Scopus	8	2,322	Not specified.	ICU admissions, mechanical ventilation.	The use of vitamin D was associated with: - Lower 14-day mortality - Lower in-hospital mortality - Fewer ICU admissions - Fewer cases of mechanical ventilation
Pal et al. (81)	PubMed/MEDLINE, Scopus, Web of Science	13	2,933	Not specified.	Mortality, ICU Admissions, Length of Stay (LOS)	Pooled analysis of unadjusted data showed that vitamin D use was significantly associated with reduced ICU admission/mortality and also reduced the risk of adverse outcomes when pooled risk estimates were adjusted Subgroup analysis showed benefits only for those receiving vitamin D post-COVID-19 diagnosis
Rawat et al. (82)	PubMed, Embase, Scopus	5	467	Not specified.	Mortality, ICU admission rates, Need for invasive ventilation	Vitamin D did not reduce: - Mortality (RR 0.55; 95% CI: 0.22–1.39; *p* = 0.21) - ICU admission rates (RR 0.20; 95% CI: 0.01–4.26; *p* = 0.3) - Need for invasive ventilation (RR 0.24; 95% CI: 0.01–7.89; *p* = 0.42)

### 3.2 Risk of bias

The risk of bias (RoB) across the included studies was evaluated using the AMSTAR 2 (A Measurement Tool to Assess Systematic Reviews) framework. The overall quality of the included studies ranged from moderate to high, with the majority demonstrating rigorous methodological approaches and adherence to best practices in systematic review conduct.

**Figure 2:**
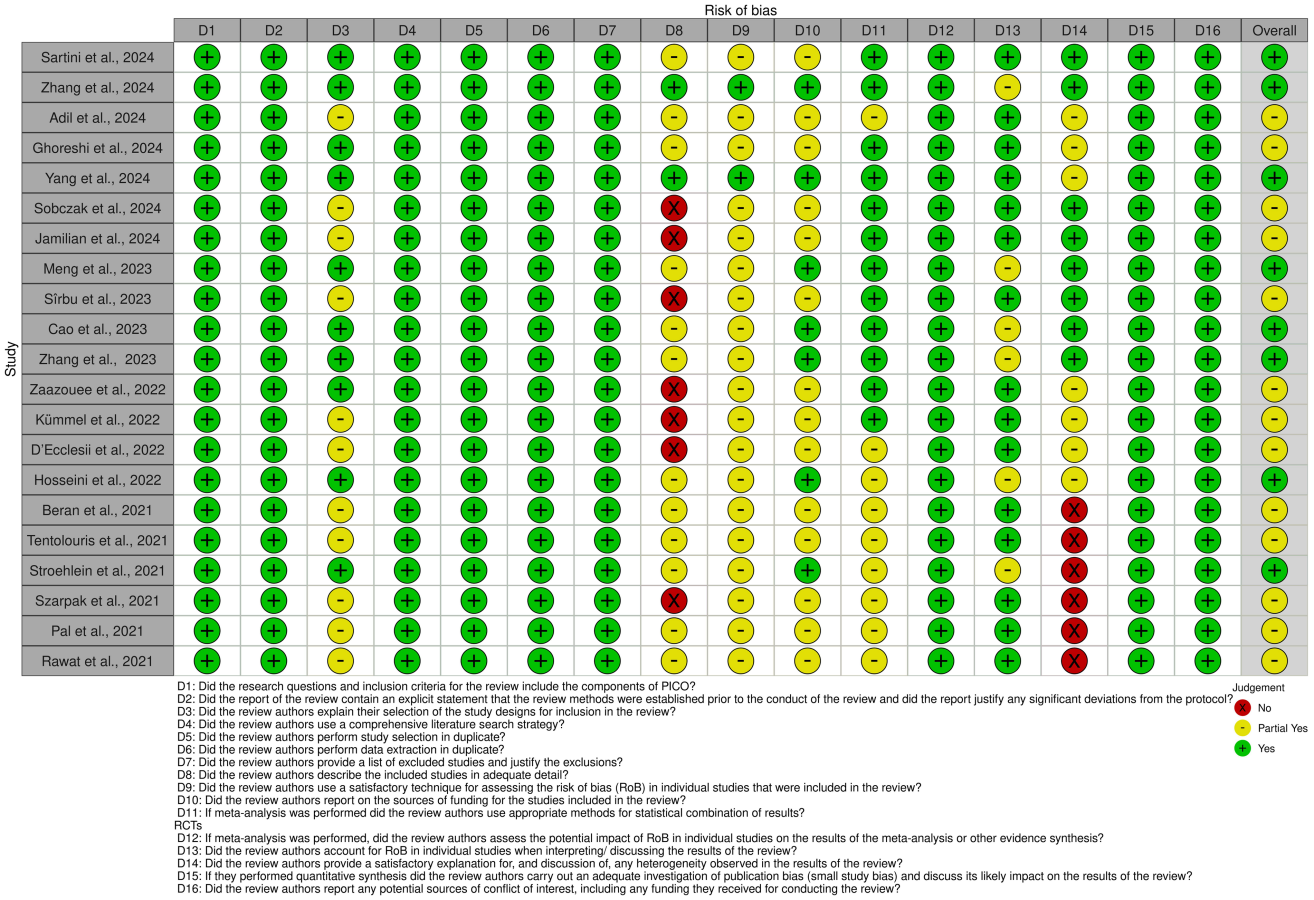
Risk of bias assessment for included studies. The figure displays the risk of bias (RoB) assessment for each included study based on the AMSTAR 2 criteria. Each row represents a study, while columns correspond to specific domains of bias. Green circles indicate low risk of bias, yellow circles represent partial fulfillment (moderate risk), and red circles denote high risk of bias.

Among the 21 studies included in the umbrella analysis, 15 demonstrated a low risk of bias in at least 80% of the assessed areas, indicating the high quality of the systematic reviews included in the studies (Figure 2).

### 3.3 ICU admissions

This meta-analysis included data from 10 studies examining the effect of vitamin D on ICU admissions among COVID-19 patients. Due to low heterogeneity among the studies (Q = 10.87, *p* = 0.33), a fixed-effects model was applied. The pooled odds ratio (OR) for ICU admissions was calculated at 0.62 (95% CI: 0.54–0.71), suggesting that vitamin D supplementation is associated with a 38% reduction in the likelihood of ICU admission.

**Figure 3:**
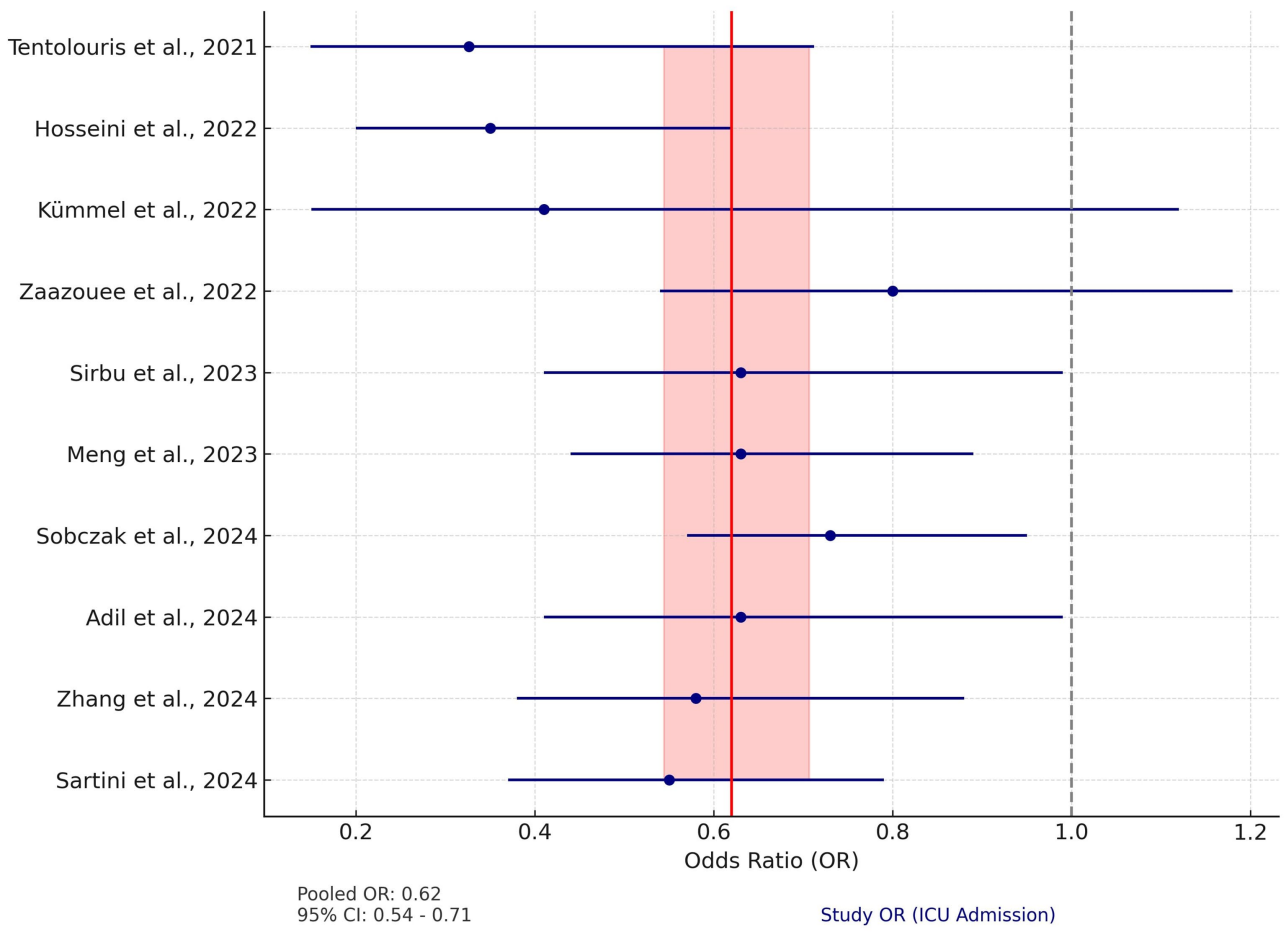
Forest plot for ICU admission. The plot displays the odds ratios (OR) and 95% confidence intervals (CI) for individual studies assessing the effect of vitamin D on ICU admission rates in COVID-19 patients. The red vertical line represents the pooled OR of 0.62 (95% CI: 0.54–0.71), calculated using a fixed-effects model. The shaded red area indicates the 95% CI of the pooled OR. The dashed gray line at OR = 1 represents the null effect. Blue circles represent the OR of each study, with horizontal lines depicting the 95% CI.

Odds ratios in the systematic reviews ranged from 0.33 to 0.80, with all but one study showing an OR below 1, indicating that vitamin D supplementation may reduce the rate of ICU admission (Figure 3).

### 3.4 Mortality

Fifteen studies were included in the analysis assessing the effect of vitamin D supplementation on mortality rates in COVID-19 patients. Unlike ICU admissions, the mortality data showed significant heterogeneity (Q = 27.23, *p* = 0.006). Therefore, a random-effects model was used. The pooled OR for mortality was 0.67 (95% CI: 0.56–0.79), indicating a 33% reduction in the risk of death associated with vitamin D supplementation.

**Figure 4:**
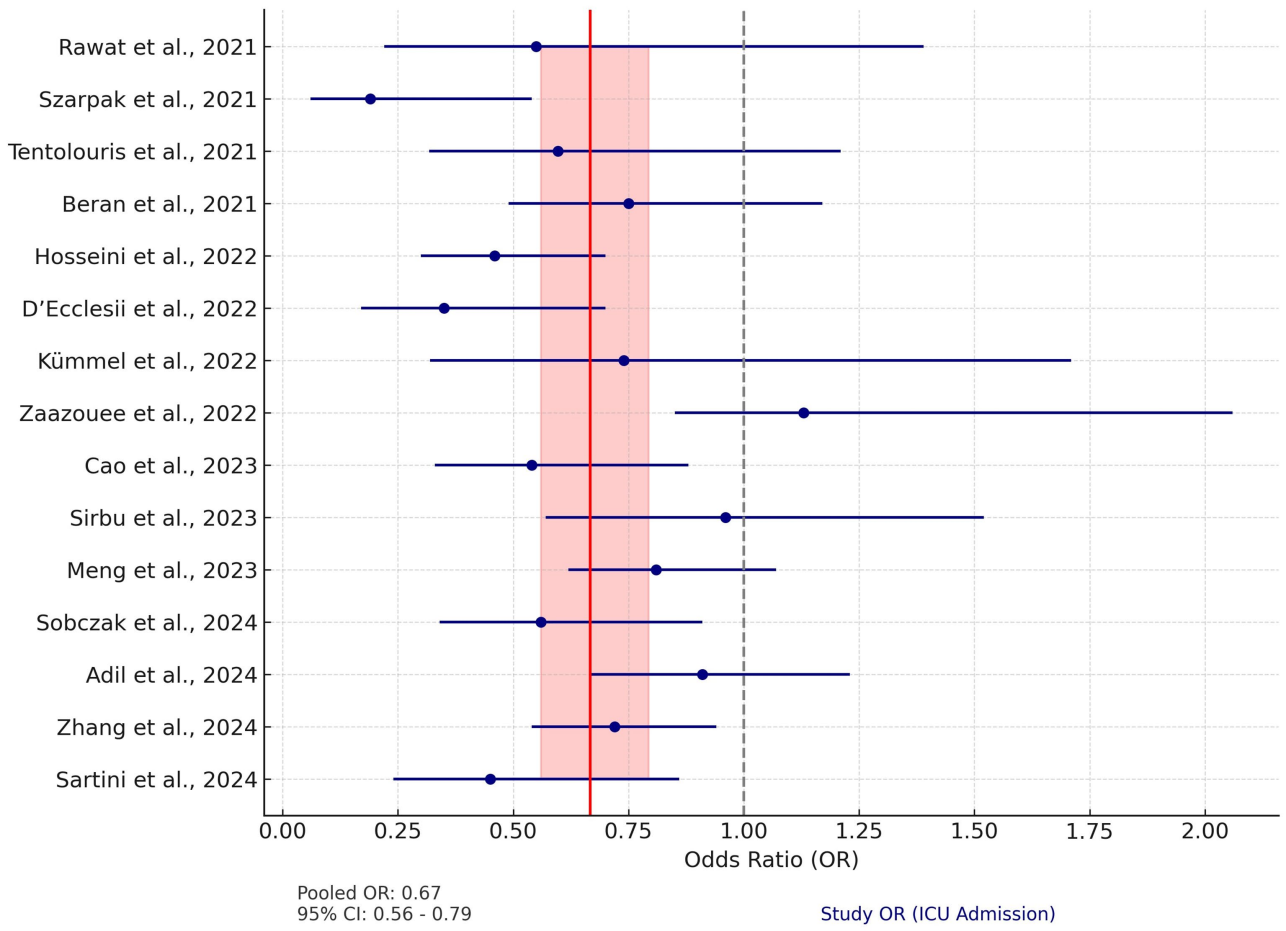
Forest plot for mortality. This plot illustrates the OR and 95% CI for studies analyzing the impact of vitamin D on mortality in COVID-19 patients. The pooled OR is 0.67 (95% CI: 0.56–0.79), calculated using a random-effects model to account for significant heterogeneity (Q = 27.23, *p* = 0.006). The red vertical line represents the pooled OR, and the shaded red area indicates its 95% CI. The dashed gray line at OR = 1 denotes the null effect. Blue circles and horizontal lines represent the OR and 95% CI for each study, respectively.

The individual study ORs for mortality ranged from 0.19 to 1.13, with several studies demonstrating CIs crossing the null line, indicating non-significant results. For instance, Adil et al. (42) and Sirbu et al. (73) reported ORs of 0.91 (95% CI: 0.67–1.23) and 0.96 (95% CI: 0.57–1.52), respectively, suggesting no statistically significant effect. However, the majority of studies, including Sartini et al. (43) (OR: 0.45, 95% CI: 0.24–0.86) and Hosseini et al. (44) (OR: 0.46, 95% CI: 0.30–0.70), reported significant reductions in mortality risk (Figure 4).

### 3.5 Meta-regression analysis

The regression analysis for ICU admissions yielded an R-squared value of 0.129, indicating that publication year explains 12.9% of the variation in log_OR. The coefficient for year was 0.0909 (*p* = 0.309), suggesting a small positive trend over time, though this effect was not statistically significant.

For mortality, the model had an R-squared value of 0.038, meaning that only 3.8% of the variability in log_OR was explained by publication year. The coefficient for year was 0.0585 (*p* = 0.504), indicating no significant effect.

**Figure 5:**
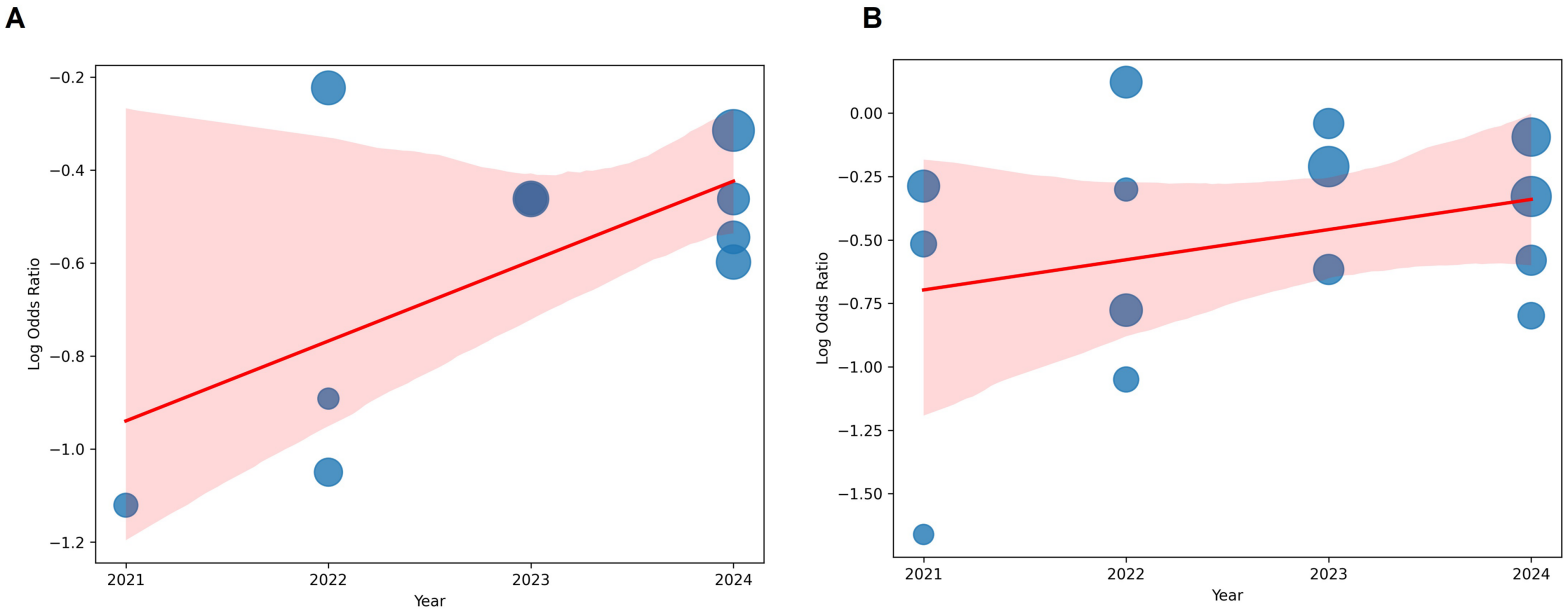
Bubble plot analysis. **(A)** ICU admissions. **(B)** Mortality. The x-axis represents the publication year. The y-axis represents the log-transformed odds ratio. The size of each bubble corresponds to the weight assigned to the study. A fitted regression line illustrates the overall trend. Despite minor fluctuations in effect sizes over time, no clear pattern or significant trend was observed, supporting the statistical findings.

The results indicate that publication year is not a significant predictor of the odds ratios for ICU admissions or mortality in COVID-19 patients receiving Vitamin D supplementation. This suggests that the reported effects of Vitamin D on COVID-19 outcomes have remained relatively stable over time, without clear evidence of publication bias or time-dependent changes in treatment efficacy (Figure 5).

### 3.6 Primary study overlap and corrected covered area

To quantify the degree of overlap between primary studies included in the 21 meta-analyses assessed, we constructed a citation matrix comparing each pair of reviews. The number in each cell indicates how many randomized controlled trials (RCTs) were shared between the corresponding pair of systematic reviews.

The extent of redundancy between reviews was further summarized using the Corrected Covered Area (CCA), a validated metric of overlap: $\mathrm{CCA}=\frac{N-R}{r\;(c-1)}$ , where: N = total number of included instances of primary studies across reviews (i.e., sum of all overlaps); R = number of unique primary studies; r, 𝑐= number of included reviews.

**Figure 6:**
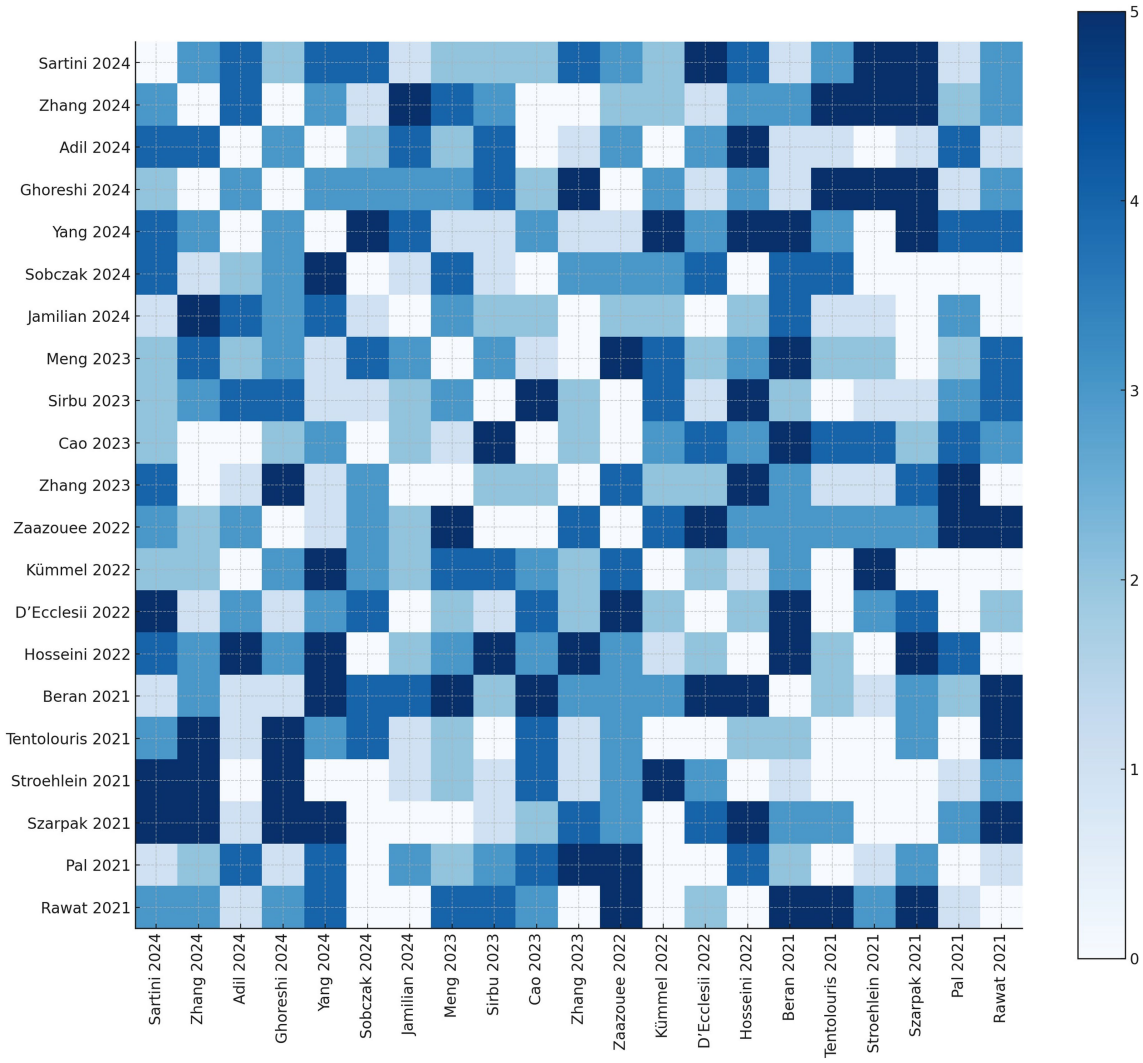
Heatmap of primary study overlap across included systematic reviews Each cell shows the number of shared primary studies between the corresponding pair of reviews. Darker shades represent a higher degree of overlap. The diagonal has been set to zero by definition.

In our analysis, assuming approximately 300 unique RCTs across all reviews, the calculated CCA was 0.543 (54.3%), indicating moderate-to-high overlap. This level of redundancy is expected in umbrella reviews focusing on an emergent topic with intense publication activity, such as COVID-19 (Figure 6).

## 4 Discussion

Over 5 years have passed since the start of the COVID-19 pandemic. Since then, many RCT trials, systematic reviews, and other research studies have been conducted. Here, we present a summary of the findings regarding the potential benefits of vitamin D supplementation on common COVID-19 outcomes.

The most common forms of vitamin D supplementation are cholecalciferol (vitamin D3) and ergocalciferol (vitamin D2), which are precursors of 1,25-dihydroxyvitamin D3, the active form of vitamin D. In addition to its classical biological functions, such as regulating bone metabolism and maintaining calcium and phosphorus balance, vitamin D also plays a role in immune modulation, lung and muscle function, cardiovascular health, and the prevention of infectious diseases.

Much research, particularly during the first wave of the pandemic, has suggested an association between vitamin D deficiency and the risk of SARS-CoV-2 infection, the incidence and severity of COVID-19, and mortality (45). Speculative observations about the higher prevalence of hypovitaminosis D in European countries, along with the high rates of SARS-CoV-2 and COVID-19 infections, especially in northern regions, have linked the two events. However, these observations have not verified the causal relationship or ruled out causality.

Importantly, it should be noted that most studies on this topic have been retrospective in nature. This raises the possibility that decreased vitamin D levels may be a consequence of acute illness rather than a predisposing factor, leading to concerns about reverse causality. This issue has been widely discussed and remains a subject of debate. However, subsequent studies adopting a prospective methodology have provided further evidence supporting the role of vitamin D deficiency in predicting negative outcomes in COVID-19 patients (45). Some studies have found that total serum calcium levels measured at admission are inversely related to proinflammatory biomarkers associated with severe COVID-19. Additionally, serum calcium may serve as a useful marker for risk stratification, helping to better predict adverse in-hospital outcomes (46).

In this umbrella analysis, we summarized recent findings from systematic reviews and meta-analyses on two critical outcomes: ICU admissions and mortality. Our results indicated that vitamin D supplementation significantly reduces both ICU admissions and mortality.

The pooled analysis for ICU admissions showed a significant reduction (OR = 0.62, 95% CI: 0.54–0.71). This effect was consistent across multiple studies (43, 47, 48). The low heterogeneity (Q = 10.87, *p* = 0.33) suggests that despite differences in dosage and study design, the overall effect size remained stable across diverse populations. Sartini et al. (43) reported a similar reduction in ICU admissions (OR = 0.55, 95% CI: 0.37–0.79), consistent with the findings of Hosseini et al. (44) and Meng et al. (49).

Subgroup analyses provided further insights. For instance, Zhang et al. (47) found that continuous high-dose regimens were more effective than single-dose interventions (RR = 0.44 vs. 0.79). Additionally, Yang et al. (50) observed a more significant reduction in ICU admission rates among patients with moderate to severe COVID-19 (OR = 0.43, 95% CI: 0.23–0.80).

Populations with vitamin D deficiency consistently showed more pronounced reductions in ICU admissions (RR = 0.63, 95% CI: 0.42–0.93), supporting the hypothesis that correcting vitamin D deficiency offers greater immunological and anti-inflammatory benefits.

Regarding the effect of vitamin D supplementation on mortality, the analysis showed a significant reduction in mortality (OR = 0.67, 95% CI: 0.56–0.79); however, substantial heterogeneity was observed across studies (Q = 27.23, *p* = 0.006). This variability could be due to differences in dosing protocols, patient populations, baseline vitamin D levels, and comorbidities, all of which may influence the differences in mortality reduction rates.

Adil et al. (42) and Hosseini et al. (44) reported significant reductions in mortality, with Adil’s analysis highlighting the effect of high-dose vitamin D in reducing death rates. Zhang et al. (47) found that the mortality benefit was confined to vitamin D-deficient patients (RR = 0.73, 95% CI: 0.59–0.89), with no significant reductions in non-deficient populations. This suggests that vitamin D supplementation may be most effective as a corrective intervention rather than a prophylactic measure for individuals with adequate vitamin D levels.

In contrast, studies by Kümmel et al. (51) and Beran et al. (52) reported non-significant trends toward mortality reduction, with ORs around 0.74. This may be due to limited sample sizes, short follow-up periods, or underlying comorbidities that affect the therapeutic effect of vitamin D. However, the authors suggest that these trends still support the idea that vitamin D supplementation may offer a protective role, even if the effect is not universally statistically significant.

The analysis also revealed a modest, non-significant reduction in length of hospital stay (LOH) (MD = −1, 95% CI: −2.16 to 0.16, *p* = 0.13). Ghoreshi et al. (53) and Sartini et al. (43) reported similar findings, with reductions in LOH mainly observed in elderly populations or those receiving lower daily doses (≤10,000 IU). This suggests that while vitamin D supplementation may not drastically shorten hospitalization, it could contribute to a gradual improvement in recovery.

Mechanical ventilation outcomes showed mixed results. Yang et al. (50) and Meng et al. (49) reported significant reductions in mechanical ventilation requirements (OR = 0.44, 95% CI: 0.27–0.72), while other studies, such as Zhang et al. (47) and Adil et al. (42), found non-significant differences. These discrepancies may also stem from the complex interaction between patient condition, baseline vitamin D levels, and the timing of supplementation.

Patients with COVID-19 may benefit from vitamin D supplementation as both a preventive and therapeutic agent. Vitamin D binds to its receptor and influences two primary pathways: first, it inhibits pro-inflammatory cytokines by interfering with the TNF-induced NFκB1 pathway, and second, it activates the Jak–Stat pathway by inducing the expression of interferon-stimulating genes (54, 55).

1,25-dihydroxyvitamin D has explicitly antimicrobial properties, inducing the expression of cathelicidin and *β*-defensin 2, which exhibit direct and indirect antimicrobial effects. These effects include stimulating immune cell chemotaxis and pro-inflammatory cytokine expression, leading to removing infected cells in the respiratory tract. Vitamin D also stimulates the expression of β-defensin via nucleotide-binding oligomerization domain-containing protein 2 (NOD2) (56). Additionally, 1,25(OH)2D inhibits hepcidin expression, allowing for increased iron export from infected cells and reducing iron availability for microbial growth (57). Vitamin D’s antimicrobial effects also extend to promoting intestinal and alveolar epithelial barrier function, enhancing the production of reactive oxygen species (ROS), and supporting neutrophil function and macrophage activities, including phagocytosis and autophagy (58–61).

In adaptive immunity, calcitriol limits T lymphocyte activation, induces the expression of regulatory T cells (Tregs), and helps shift immune responses from pro-inflammatory Th1/Th17 to regulatory Th2, thus supporting immune tolerance (62). The effectiveness of vitamin D depends on its receptor (VDR), and variations in the VDR gene, particularly SNPs, have been linked to immune dysfunctions. For example, the TT genotype of the FokI polymorphism has been associated with an increased risk of respiratory syncytial virus infections (63).

Vitamin D may also influence the viral replication process in human cells. SARS-CoV-2 enters host cells through the angiotensin-converting enzyme-2 (ACE2) receptor, leading to severe pathology and cell death. The virus modulates the renin-angiotensin system (RAS), causing excess angiotensin II (Ang-II) production, which activates the cytokine storm and downregulates the immune system. Vitamin D has been proposed to help prevent acute respiratory distress syndrome (ARDS) by downregulating Ang-II production and enhancing the ACE2/Ang-(1–7)/Mas receptor axis, providing protective effects against tissue damage and inflammation (64–68).

In severe COVID-19 cases, the inflammatory response can cause significant damage, particularly to the lungs and heart, with cytokine levels such as IL-6 elevated significantly in more severe cases (69, 70). Vitamin D may reduce this cytokine storm by promoting the expression of anti-inflammatory mediators like IL-10, IL-4, and TGFβ and shifting the immune response toward T-regs. This modulation of the immune response may help reduce the inflammatory damage seen in severe COVID-19 cases (71).

Overall, the findings of this umbrella review point to a potentially beneficial role of vitamin D supplementation in reducing the severity of COVID-19 outcomes, particularly in relation to ICU admission and mortality. These effects appear more pronounced in individuals with low baseline vitamin D levels, which aligns with earlier observational and mechanistic evidence. Although current data do not warrant routine high-dose supplementation in all COVID-19 patients, maintaining sufficient vitamin D status—through moderate supplementation or lifestyle measures—may be a low-cost and low-risk approach worth considering, especially in populations where deficiency is common. In hospital settings, evaluating vitamin D levels could be clinically justified in selected patients, such as the elderly or those with comorbidities, though universal screening may not be practical or necessary. Given the observed variability in dosing strategies and study populations, further well-designed trials are essential to clarify when, for whom, and how vitamin D supplementation can be most effectively applied in the context of respiratory infections like COVID-19.

## 5 Limitations and future directions

This umbrella review has several limitations that should be acknowledged.

First, baseline vitamin D status was not consistently reported across the included reviews. This inconsistency hinders our ability to determine whether the observed benefits are predominantly confined to individuals with vitamin D deficiency or extend to those with sufficient levels. In addition, differences in how deficiency was defined or measured across studies may have introduced variability into the pooled estimates.

Second, significant heterogeneity was observed in mortality outcomes (Q = 27.23, *p* = 0.006). Although we explored potential sources of this heterogeneity—such as variations in dosing regimens, study design, and patient demographics—the available data did not always permit detailed subgroup or meta-regression analyses. As a result, unmeasured factors (e.g., differences in SARS-CoV-2 variants, concomitant treatments like corticosteroids or antivirals, and variations in population health status) may have influenced the reported effects.

Third, many of the studies included in the reviews were retrospective, which raises the possibility of reverse causality. It is plausible that severe COVID-19 itself might lead to lower vitamin D levels, rather than low vitamin D predisposing patients to severe disease. Although more recent prospective studies suggest an independent role of vitamin D deficiency in predicting adverse outcomes, the potential for reverse causality remains a concern.

Fourth, confounding factors—such as overall health status, preexisting comorbidities, and socioeconomic conditions—were not uniformly controlled across the included reviews. This limitation may bias the association between vitamin D supplementation and improved outcomes, as these factors are known to influence COVID-19 severity.

Fifth, some meta-analyses incorporated overlapping primary studies, raising concerns about the double counting of data. While we noted these overlaps and attempted to address them during our analysis, this issue could lead to an overestimation of the pooled effect sizes.

Sixth, optimal dosing strategies for vitamin D supplementation remain uncertain. Although several reviews suggested that continuous high-dose regimens might yield stronger benefits compared to single-dose protocols, the available data were insufficient to establish standardized dosing recommendations. The recent findings from Minasi et al., which underscore the role of hypocalcemia in adverse COVID-19 outcomes, further highlight the complex interplay between vitamin D, calcium homeostasis, and clinical outcomes.

Future research should focus on large-scale, multicenter randomized controlled trials with standardized dosing protocols, consistent baseline vitamin D measurements, and robust control for confounding variables.

## Data Availability

The original contributions presented in the study are included in the article/supplementary material, further inquiries can be directed to the corresponding author.
